# Supplementary material for: Alcids ‘fly’ at efficient Strouhal numbers in both air and water but vary stroke velocity and angle
Source: eLife. 2020 Jun 30;9:e55774. doi: 10.7554/eLife.55774 (PMC7332295; doi:10.7554/eLife.55774)
Supplement: Supplementary file 1. — Calculated body lengths were used to convert from units of species-specific body length to metric units. Average culmen length was calculated as the mean of all values present in the Birds of North America entry (Rodewald, 2015) for adult birds (males and females) of that species. Multiple birds were digitized in some photographs. See Materials and methods for details. [file elife-55774-supp1.docx]

**Supplementary Table 1: Average culmen length, asset numbers, and calculated body length during flight for each of four species of alcid.** Calculated body lengths were used to convert from units of species-specific body length to metric units. Average culmen length was calculated as the mean of all values present in the *Birds of North America* entry (Rodewald, 2015) for adult birds (males and females) of that species. Multiple birds were digitized in some photographs. See methods for details.

| Species | Average culmen length (mm) | Asset number | Calculated body length during flight (eye-to-tail) (mm) |
| --- | --- | --- | --- |
| Common murre | 45.7 | ML130936951  ML131083411  ML133502561  ML135189951  ML135262691  ML138943951  ML153828011  ML107922131  ML109190921  ML109269361  ML110104101  ML123633261  ML124704241  ML127601661  ML128211881 | 357.2 |
| Horned puffin | 48.3 | ML128125701  ML129591491  ML132256341  ML132256341  ML133379331  ML133502351  ML135262671  ML135482301  ML103661271  ML103661271  ML110814591  ML114538841  ML114538901  ML116573881  ML128125681 | 260.7 |
| Pigeon guillemot | 34.9 | ML137281651  ML137369751  ML100083281  ML102575791  ML107512881  ML107512911  ML109212741  ML109212751  ML109270431  ML109982411  ML116574131  ML126682601  ML126741691  ML129421451  ML129421471 | 276.7 |
| Tufted puffin | 58.2 | ML149438221  ML77583491  ML103948801  ML108569631  ML108569931  ML108988301  ML110239891  ML110391621  ML111384021  ML112301331  ML113722471  ML120413871  ML131728531  ML133386421  ML135063271 | 290.4 |

**Rodewald, P. (Editor)** (2015). The Birds of North America: https://birdsna.org. *Cornell Lab. Ornithol. Ithica, New York*.
